# Substance of Lectures on Gold

**Published:** 1878-08

**Authors:** 


### ARTICLE V.

### *Substance of Lectures on Gold.*—Continued.

BY PROF. HODGKIN.

Refining.—We have seen, in previous lectures, that it is of the utmost importance that our gold, at least for certain uses, should be absolutely pure, and approximately so for any use connected with dental mechanism. If it be a fact that so small proportion of some of the baser metals as has been stated, is sufficient to render gold unmalleable, it is of extreme importance that we should keep these from our gold, and if accidentally contaminated thereby, of equal importance that we should possess some means of eleminating these impurities. Such knowledge cannot come amiss to us if only on rare occasions we have use for it, and if, as we all hope, the time is coming when gold plates will be more commonly used than at present, our knowledge of its manipulation cannot fail to be of service to us.

Reverting to the previous lectures, you will remember that we made two general divisions of the dental scrap found about our offices. One, in which the impurities were only mechanically mingled with the gold, the other in which they were melted or alloyed into its substance. It will readily occur to you that in the first case methods may be used for removing extractions substances which would be ineffectual in the other. We have only to apply a magnet for example to the mass, and thus remove any particles of iron, and we have only to use some solvent of copper, tin, lead, &c.,—something which, while dissolving them, does not dissolve the gold, to reduce these to a state of solution, that they may then be washed out. Our knowledge of chemistry can be made available here, and indeed you will find that this study, which some of you seem to think is rather out of place in practice, and dry study under any circumstances, is one of the most useful and entertaining of any with which you have to deal. It is important to know what acids act on certain metals, and the result of their action, and also what is the result of the combination of certain acids. If, for example, we wished to remove iron by another means than the magnet—and I advise other means for the reason that particles of gold are removed along with the fragments of iron—we should know the most ready solvent of iron, and also the product of solution, and the steps necessary to remove the solution from the gold. Again, we find that where lead and tin and zinc are present, it is not a matter of indifference which one of the acid solvents of those metals we use, and it is necessary also to know what will dissolve these. Zinc and iron are acted on by hydrochloric acid, and the salts of these medals are soluble, and this acid will act likewise on the silver, if any be present; but while the first-named salts are soluble and may be washed out with clear water, the silver solution is insoluble in the water, and so is as much in one way as if it had not been attacked at all. Again, while some metals, as iron, zinc, are attacked by sulphuric acid, this acid will not, at least in anything like the dilute state in which we use it, attack lead. It is important then that this part of chemistry be well understood by us, else each step is in a fog; and in operations involving such a precious metal, as the one we are now dealing with, accuracy is essential; accuracy both of knowledge and manipulation. May I say again that *theory* must precede *practice*; we must know before we can do, and the man who depends on his practical knowledge is, when he leaves the ground of that knowledge, helpless, except so far as he may blunder and stumble on the things he wants. I tell you that he who is fully armed with scientific knowledge is superior to chance, and is able to hold the reins of nature's goings.

Nitric Acid ($N_2O_5$) is perhaps our most convenient acid in removing the impurities that are mechanically mingled with the gold. It acts on all the metals except the gold and platinum,—that is of those we are likely to find in our present search; understanding that we are now only seeking to get rid of the metals, not melted or alloyed with that we are seeking to purify. The action of the acid may be greatly hastened by heat, using a sand-bath for this purpose. Care should be taken that no hydrochloric acid is present, as this mixture acts on the gold, as we will see further on.

Two general methods here claim our attention for the purification of gold; one partial and imperfect, but largely useful to jewellers and others, and to some extent useful to us; the other perfect, exact, a strictly chemical process, giving us absolutely pure gold. The first is that of refining by fire, or the "dry process," as it is usually called; the other is refining by acids, or the "humid process." By means of the first, which involves only the use of a crucible and a furnace, and requires only the use of a few simple reagents, we are enabled to get rid of all the base metals, but retain the platinum and silver, if these are present; while by the other, every metal can be removed and the gold left in its pure state, ready for the foil-beater. We will take up the first method and briefly describe it. Its success depends on the principle that bare metals are oxydizable, while the noble metals are not; and the process is simply that of melting the mass of alloy in a suitable crucible and oxydizing the impurities, if susceptible of this.

Any ordinary stove will answer our purpose. We find sufficient heat in the office or parlor furnace, and if it be summer time a small preserving furnace will answer. The small forges which are made for this and for other purposes, and which are found in the back shop of almost every jeweller, answer well; and the apparatus recently designed by Mr. Fletcher, of England, is doubtless excellent. With a good blow-pipe a considerable mass of gold may be melted on a piece of charcoal, and although this substance is inadmissible for this purpose, in the process under consideration, a substitute can be readily made for it by carving out a cavity in a piece of carbon from the gas retort, or making a shallow dish of plumbago, or even of sand and plaster, though there may be some risk of cracking in the last case. Molten gold when let fall, scatters into exceedingly minute particles, and the probabilities are that little of it would be recovered if spilled. For any mass over one-fourth of an ounce it would be best to trust the crucible and furnace. The crucibles are (1) Plumbago and (2) Hessian, so-called, for most of those we see for sale at the depots, are made in this country,—and are excellent. Those made of plumbago are best, as they will stand any amount of heat, and it is said bear frequent reheatings with little risk of cracking, which cannot be said of those made of sand and clay. For ordinary dry refining however the Hessian crucibles are most convenient, as it will be found advantageous to break the crucible after cooling, the accumulation of borax and other substances on the surface rendering it difficult to pour the gold.

The choice of fuel is of some importance. Coal of a mineral origin is often contaminated with sulphur, and this element is greatly in the way in our work, making the gold brittle and unmalleable. Bituminous coal, unless made into coke is inadmissible. A clear fire of anthracite is excellent; it is well to let it burn for a while before using it, that any sulphur fumes may pass off. Charcoal is best per haps, as being free from any suspicion of sulphurous taint; that made from hard wood is best. The crackling of charcoal fires, made of pine and chestnut, is annoying. The gun powder makers use willow charcoal, from which the bark has been removed previously to burning.

Whatever fuel is used it is well to see that the crucible sets firmly in the midst, and that it is well supported at the bottom. A solid lump of charcoal is useful for this, setting the crucible on it; or it may be set on the bottom of an inverted crucible. A pair of suitable tongs for handling the crucible are necessary adjuncts of the furnace. The fuel should be placed well around the crucible, as in pouring the metal it is of importance that the vessel should be hot all the way to the top. If the gold comes in contact with the lip of the crucible, with this last at less than red heat, it is instantly chilled, and its flow in part arrested.

A glass or porcelain rod—the latter is best—is useful to stir the molten mass, and bring the impurities successively to the surface for exposure to the reagents. An iron rod well coated with borax, by heating it in contact with this substance, will answer.

The reagents are used with a view to their chemical action, the destruction of their combinations, the liberation of their constituent elements and the action of the nascent oxygen, chlorine, &c., on the substances it is designed to eleminate from the mass. A knowledge of the chemistry of this process is so interesting that we will take it up in a separate lecture.

